# DNMT1/miR-152-3p/SOS1 signaling axis promotes self-renewal and tumor growth of cancer stem-like cells derived from non-small cell lung cancer

**DOI:** 10.1186/s13148-024-01663-5

**Published:** 2024-04-15

**Authors:** Qing Yuan, Rubo Wang, Xiang Li, Fei Sun, Jiazhi Lin, Zhimin Fu, Jiansong Zhang

**Affiliations:** 1 Department of Preclinical Medicine, Medical College, Hunan Normal University, Changsha, 410013 China; 2 Key Laboratory of Study and Discover of Small Targeted Molecules of Hunan Province, Changsha, 410013 China; 3 Department of Pathology, Hunan Cancer Hospital, The Affiliated Cancer Hospital of Xiangya School of Medicine, Central South University, Changsha, 410013 China; 4 Department of Gynaecology and Obstetrics, Shenshan Medical Center, Memorial Hospital of Sun Yat-sen University, Shanwei, 516500 Guangdong China; 5 Department of Thoracic Surgery, The Tenth Affiliated Dongguan Hospital, Southern Medical University (Dongguan People’s Hospital), Dongguan, 523000 China

**Keywords:** DNA Methyltransferase 1, Cancer stem cells, Non-small cell lung cancer, miR-152-3p, SOS1

## Abstract

**Background:**

CSLCs(Cancer stem cell-like cells), which are central to tumorigenesis, are intrinsically influenced by epigenetic modifications. This study aimed to elucidate the underlying mechanism involving the DNMT1/miR-152-3p/SOS1 axis in regulating the self-renewal and tumor growth of LCSLCs (lung cancer stem-like cells).

**Materials and methods:**

Target genes of miR-152-3p were predicted using TargetScan Human 8.0. Self-renewal and tumor growth of LCSLC were compared in suspension-cultured non-small cell lung cancer (NSCLC) cell lines H460 and A549 cell-derived globe cells. Functional effects of the DNMT1/miR-152-3p/SOS1 axis were assessed through gain-of-function experiments in vitro and in vivo. Additionally, luciferase reporter assays were employed to analyze the interaction among DNMT1, miR-152-3p, and SOS1.

**Results:**

Our findings highlight a negative interaction between DNMT1 and miR-152-3p, resulting in reduced miR-152-3p level. This, in turn, leads to the alleviation of the inhibitory effect of miR-152-3p on the target gene SOS1, ultimately activating SOS1 and playing an essential role in self-renewal and tumor growth of LCSLC. However, the alteration of SOS1 does not affect DNMT1/miR-152-3p regulation. Therefore, it is reasonable to infer that the DNMT1/miR-152-3p negative feedback loop critically sustains self-renewal and tumor growth of LCSLC through SOS1.

**Conclusions:**

This study reveals a novel mechanism underpinning self-renewal and tumor growth of CSLC (cancer stem cell) in NSCLC and identifies potential therapeutic targets for NSCLC treatment.

**Supplementary Information:**

The online version contains supplementary material available at 10.1186/s13148-024-01663-5.

## Introduction

Non-small cell lung cancer (NSCLC) accounts for over 80% of all lung cancer cases, making it the deadliest malignancy globally [1]. While current treatment modalities can alleviate symptoms, the 5-year overall survival rate of NSCLC remains below 20% [2]. Therefore, novel therapeutic targets are urgently needed to enhance the efficacy of NSCLC treatment. Recent studies have placed considerable emphasis on CSCs or CSLCs [3–5]. CSLCs constitute a small subset of tumor cells derived from bulk tumors, possessing stem-like features [6–8]. Currently, researchers primarily enrich or identify CSLCs using cell surface markers such as CD133 and CD44 [9], pluripotent transcription factors such as Oct4 and Sox2 [10, 11], or sphere formation assays conducted in CSC medium [12–14]. It is imperative to thoroughly investigate the specific developmental mechanisms of CSLC, develop new targeted drugs, and uncover novel therapeutic targets.

Epigenetics plays a key role in regulating the progression, invasion, and metastasis of tumor growth [15–17]. Numerous studies have underscored the close association between DNMT1 and the development and amplification of multiple tumors. For instance, DNMT1 affects cell proliferation in NSCLC by promoting the methylation of downstream molecular promoters [18]. It also facilitates the growth and metastasis of breast cancer [19]. Moreover, DNMT1 is implicated in the upregulation of the lung cancer stem-like cell (LCSLC) population [20]. In this study, we aimed to elucidate the specific mechanism underlying the role of DNMT1 in NSCLC initiation and progression.

MicroRNAs can engage various regulatory pathways during different stages of cell differentiation, growth, and apoptosis [21]. They hold a critical role in CSCs [22] and serve as valuable markers for accurately predicting the prognosis of lung cancer [23]. Notably, miR-152 stands out as a promising biomarker for detecting NSCLC [24] and regulating its metastasis [25]. DNA methylation affects the expression levels of numerous miRNAs in several tumors. The high expression of DNMT1 leads to miR-152 promoter methylation, which downregulates the expression of miR-152. By contrast, the low expression of miR-152 upregulates the expression level of DNMT1, resulting in DNMT1-mediated DNA promoter methylation and the development of a negative DNMT1/miR-152 loop [26–28]. Recently, biguanides have been shown to modulate DNMT1/miR-152 expression in lung cancer [29]. However, the precise mechanism governing the self-renewal and tumor growth of LCSLCs through the interaction of DNMT1 and miR-152 remains unclear.

The guanine nucleotide exchange factor SOS1 plays a critical role in modulating drug sensitivity in gastroesophageal cancer [30]. Additionally, miR-152 and miR-148, being part of the same gene family [29], have been shown to disrupt various signaling pathways involving SOS1 and SOS2 proteins in B cells [31]. The potential for enhancing the efficacy of multiple tumors lies in the modulation of SOS1 expression [32–34]. However, further investigations are required to elucidate how the reduced expression of miR-152-3p and the subsequent overexpression of SOS1 within the context of DNMT1 epigenetic regulation may contribute to the enrichment of LCSLCs and the induction of CSLC features. Understanding the pathogenesis of this novel epigenetic pattern dysregulation in self-renewal and tumor growth of CSLC will aid in addressing the challenges in the cutting-edge field of NSCLC prevention and treatment.

We aimed to demonstrate that the DNMT1/miR-152-3p negative pathway upregulates SOS1 expression in NSCLCs to induce self-renewal and tumor growth in CSLCs.

## Materials and methods

### Cell culture and CSLC enrichment

We procured the H460 cell lines, human large-cell lung cancer (LCLC) cell lines, which are a type of NSCLCs, from Shanghai Zhongqiao Xinzhou Biotech (Shanghai, China). We obtained the A549 cell lines, human lung adenocarcinoma (LUAD) cell line, which are a type of NSCLCs, from Wuhan Priscilla (Wuhan, China). The human BEP2D cell lines, human normal bronchial epithelial cell line, were obtained from Wuhan Otwo Biotech (Wuhan, China). All cell lines were authenticated and validated using the short tandem repeat (STR) method and mycoplasma testing. DNA was extracted using Axygen's Genome Extraction Kit and was amplified using a 20-STR amplification protocol in ABI 3730XL. We detected the STR locus and sex gene amelogenin on the transmission analyzer. These cells were cultured in DMEM supplemented with 10% FBS under 5% CO_2_ at 37 °C. The DMEM was purchased from Gibco and Invitrogen Life Technologies Ltd., which also supplied trypsin EDTA, fetal bovine serum, and penicillin–streptomycin double antibodies. Stem-cell culture medium (DMEM/F12, including 2% B27, 1% penicillin/streptomycin, bFGF (20 ng/mL), EGF (20 ng/mL), insulin (4 µg/mL) was used. The CSLCs of NSCLC were obtained using ultra-low adhesion six-well plates and stem-cell culture medium. We planted 1000 cells per well in suspension culture; the cells were cultured for 2 weeks and the media was added every alternate day.

## Bioinformatics analysis

Expression of DNMT1 and SOS1 in normal and NSCLC tumor tissues was analyzed using TCGA database via UALCAN (https://ualcan.path.uab.edu/analysis.html), [35] with a focus on cancer progression and lymph node metastasis.The correlation between DNMT1 and SOS1 in NSCLC was further analyzed using the GEPIA database[36] (http://gepia.cancer-pku.cn) and the Pearson correlation coefficient.The latest use of the UALCAN and GEPIA databases is dated August 28, 2023.

## DNMT1 activity detection

We next sought to determine DNMT1 activitiesin H460 and A549 cells as well as respective LCSLCs. The activities of DNMT1in nuclear extracts prepared from 2 × 10^6^cells with the EpiQuik™nuclear extraction kit (EpiGentek Group, USA) were no-nradioactively assessed with the DNMT activity/inhibition assay kit (EpiGentek Group), based on the manufacturer's instructions.Relative DNMT1 activity was normalized to that of the H460 and A549 cells, or LCSLCs. Three independent assays were carried out.

## RT-qPCR

Total RNA was isolated from H460 and A549 cells or corresponding LCSLCs or BEP2D (1 × 10^5^ cells) using the TRIzol reagent (Invitrogen; Thermo Fisher Scientific, Inc.). We performed reverse transcription with 2 μg of total RNA using the SureScript™ first-strand cDNA synthesis kit (GeneCopoeia, USA). Amplification was performed with the BlazeTaq™ one-step SYBR green real-time quantitative reverse transcription-PCR kit (GeneCopoeia) on a CFX connect fluorescent quantitative PCR analyzer (Bio-Rad, USA) at 95 ℃ (10 min), followed by 40 cycles at 95 ℃ (30 s), 55 ℃ (30 s), and 70 ℃ (30 s). We adopted the 2^−ΔΔCt^ method for data analysis, utilizing glyceraldehyde-3-phosphate dehydrogenase or U6 for normalization in three independent experiments. All primer sequences used are listed in Additional file 2: Table 1.

## Lentivirus infection and miRNA transfection

LV-15 (pGLVH1/RFP/Puro) lentiviral vectors(Hanheng,China). carrying shRNA targeting human DNMT1, LV8N (EF-1αF/mCherry/Puro), and DNMT1 plasmids were obtained from Hanheng Pharma. SOS1 cDNA lentiviral vector (pEZ-Lv201) obtained from Hanheng Pharma.For miR-152 modulation, MicrON™ miR-152 mimic and micr*OFF*™ miR-152a inhibitor, along with their negative controls, were obtained from RiboBio (Gaungzhou,China). All the sequences are shown in S Table 1.These were transfected into H460 and A549 cells using iboFECT™ CP (RiboBio) at a concentration of 50 nM, following the manufacturer’s instructions.

**Table 1:** Potential miRNAs targeting DNMT1 and SOS1 3'UTR

A				
Position 58–65 of DINMT1 3' UTR	Context + + score	Context + + score percentile	]',	Predicted relative K_u_
hsa-miR-152-3p	− 0.42	98	0.44	− 6.161
hsa-miR-l48b-3p	− 0.42	98	0.44	− 5.798
hsa-miR-l48a-3p	− 0.42	98	0.44	− 5.612

B				
Position 58–65 of SOS1 3^1^ UTR	Context + + score	Context + + score percentile	Per	Predicted relative K_p_
hsa-miR-152-3p	− 0.32	95	0.59	− 6.994
hsa-miR-148b-3p	− 0.32	95	0.59	− 6.555
hsa-miR-148a-3p	− 0.32	95	0.59	− 6.258

## Human lung carcinoma tissues

We obtained lung carcinoma tissue samples from the Hunan Provincial People’s Hospital. The samples were obtained from 25 patients, including 15 men and 10 women; they consisted of 10 cases of large cell lung cancer, 8 cases of LUAD, and 7 cases of lung squamous cell carcinoma (LUSC). We obtained the consent of all patients, and our study was approved by the Research Ethics Board at the Hunan Normal University (license number: 2023338).

## Western blot analysis

H460 and A549 cells and respective LCSLCs (5 × 10^5^cells) underwentlysis with cold RIPA buffer (Beyotime, China) supplemented with protease inhibitors. Equal amounts of total protein (50 *μ*g) underwent separation by 10% sodium dodecyl sulfate polyacrylamide gel electrophoresis (SDS-PAGE) and were transfered onto polyvinylidene fluoride (PVDF) membranes (Millipore, USA). Then, blocking with 5% skim milk was followed by overnight incubation at 4℃ with anti-α-Tubulin, anti-DNMT1, anti-SOS1, anti-CD133, anti-CD44 and anti-Oct4 and anti-Sox2 primary antibodies. Appropriate horseradish peroxidase-linked secondary antibodies (Beyotime, China) were added for 1 h at ambient. The enhanced chemiluminescence detection system (Tanon Science & Technology, China) was utilized for visualization, and antigen–antibody reaction was visualized using the ChemiDoc system (Bio-Rad, Hercules, CA, USA). Commercially sourced antibodies were used, and their details can be found in Additional file 2: Table 3. The blot intensities were quantitated using the Image J software. The data were normalized against the vehicle control using α-Tubulin antibody as a reference.

## Clonogenic assay

DMEM with 0.8% Invitrogen added was distributed to the bottom layer of a 24 well plate at a volume of 1 mL per well. Then, H460 and A549 cells (1 × 10^3^), or LCSLCs (1 × 10^3^), suspended in SCM-containing 0.4% agarose (top layer), were seeded into 24-well plates. These plates were then incubated for three weeks. After the incubation period, colonies were counted under an inverted microscope (Olympus IX53, Japan). The colony formation rate was calculated as follows: (Number of colonies formed / Number of cells seeded (1 × 10^3^ cells per well in a 24-well plate)) × 100%. The assay was carried out in triplicate.

## Spheroid Formation Assay

Prepare to use the ultra-low adhesion 24-well plate, stem-cell culture medium was added, and planted the H460 and A549 cells (1 × 10^3^), or LCSLCs (1 × 10^3^) cells per well were planted in suspension culture. The cells were cultured for 2 weeks and the media was added every alternate day. The spheroid volume was estimated as follows: *V* = (4/3)*πR*^3^. The assay was carried out in triplicate.

## Methylation-Specific PCR (MSP)

DNA was extracted from cells (1 × 10^6^) using the DNA-EZ Reagents V All-DNA-Out (Sangon Biotech, China). Genomic DNA was incubated with the Methylamp One-Step DNA Modification Kit (Epigen Tek) following the manufacturer’s instructions. PCR was carried out using HotStar Taq Polymerase (Qiagen, Germany), with methylated and unmethylated PCR primers specific to the miR-152-p promoter. The The primer sequences used for detection were provided by Sangon Biotech [37].Cycling conditions were initial denaturation at 92 °C for 3 min, 40 cycles of 92 °C for 30 s, 67 (M) or 64 °C (U) for 30 s and 71 °C for 30 s. The MSP products were visualized using 2.0% agarose gel electrophoresis.

## Luciferase reporter assay

The wild-type SOS1 3′UTR target sequence comprising the miR-152-3p binding site was inserted into the pLUC luciferase vector (Ambion, USA), following the manufacturer’s instructions. A mutated SOS1 3′UTR sequence was used as a control. These constructs (0.2 μg) were transfected into H460 and A549 cells or LCSLCs in 24-well microplates, along with miR-152-3p or miR-NC and the luciferase vector, and incubated for 48 h. Then, the dual luciferase reporter assay system (Promega) was used to assess luciferase activity following the manufacturer’s instructions.

## In vivo tumorigenicity experiments

Female pathogen-free nude BALB/c mice, aged 4–5 weeks, were procured from GemPharmatech Co., Ltd. (Chengdu, China). The mice were housed in a specific pathogen-free facility with a standard 12 h/12 h light/dark cycle and ad libitum access to regular mouse chow and water (SYXK (Xiang) 2020–0012). The study protocol was approved by the Ethics Committee of Hunan Normal University (D2022071). The female mice were assigned randomly to different experimental groups, with six mice in each group. Compared with the H460 cells and LCSLC tumor growth experiments, the H460 cells or LCSLCs were injected into the armpits of the mice. For detecting the effects of DNMT1 knockdown on the tumorigenicity of LCSLCs in vivo, the mice were injected subcutaneously with either NC-LCSLCs or shDNMT1-LCSLC suspensions to construct xenograft models. Tumor volumes and mouse weights were gauged every two days. After 21 days of treatment, the mice were euthanized using CO_2_ inhalation. The xenografts were obtained, weighed, and either snap-frozen in liquid nitrogen or fixed in 10% formalin for subsequent assays.

## Immunohistochemical staining

The tissues were fixed with 4% paraformaldehyde. The paraffin was embedded and sectioned. The sections were deparaffinized and rehydrated using xylene, followed by 100%, 95%, and 75% of ethanol. The sections were incubated with 3% H_2_O_2_ for 20 min to block endogenous peroxidase, washed with phosphate buffered solution (PBS), and boiled in Tris–EDTA retrieval solution for 5 min in a pressure cooker. Subsequently, the sections were incubated with primary antibodies overnight at 4 °C. After drying, we added secondary antibodies (GB23204, Servicebio, 1:200, Wuhan, China) to cover the tissues. They were incubated at room temperature for 50 min. The slides were washed in PBS on a destaining shaker by shaking and were spun dry. We added freshly prepared 3,3’-diaminobenzidine chromogenic solution dropwise into the circle. We performed hematoxylin counterstaining for approximately 3 min, followed by rinsing with water. The hematoxylin differentiation solution differentiated for a few seconds. After rinsing in tap water, the hematoxylin blue solution turned blue; it was rinsed with running water. After dehydration, the slices were taken out of the xylene for drying and were covered with sheets. We used antibodies against DNMT1, SOS1, and CD44 (Additional file 2 Table 4).

### FISH

The Fluorescence in situ hybridization kit (Servicebio, Wuhan, China) was used to carry out the FISH experiment. Hybridization was performed using Cy3-labeled miR-152-3p probes (CCAAGTTCTGTCATGCACTGA,500 nM,Servicebio,Wuhan, China). Subsequently, the samples were analyzed using a fluorescence microscope (Nikon, Japan).

## Statistical analysis

The SPSS 20.0 software (IBM, Armonk, NY, USA) was employed for statistical analysis. The data were expressed as mean ± standard deviation (SD). A two-tailed Student’s t-test was used for comparisons with the control groups. A one-way ANOVA with Tukey’s post hoc test was employed for pairwise comparisons among multiple groups. *P* < 0.05 was considered statistically significant.

## Results

### DNMT1, miR-152-3p, and SOS1 are potential targets in LCSLCs

**Fig. 1:**
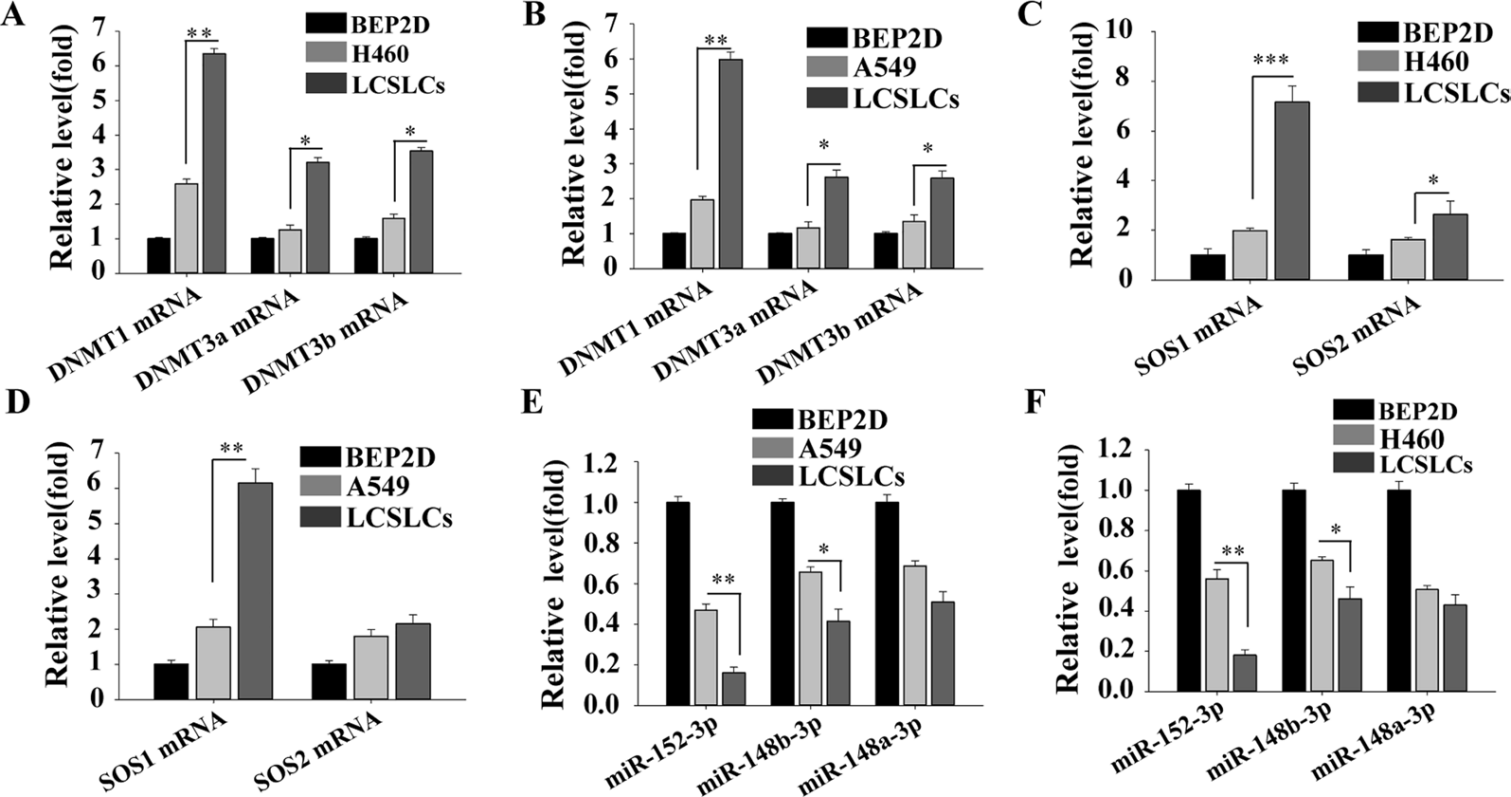
The expression of DNMTs, the miR-152/148 family, and SOSs in LCSLCs **A**, **B** Expression of DNMT1, DNMT3a, and DNMT3b mRNA in H460, A549 H460/A549-derived LCSLCs, and BEP2D cells; **C**,** D** Levels of SOS1 and SOS2 mRNA in H460, A549, H460/A549-derived LCSLCs, and BEP2D cells; **E**–**F** Levels of miR-152-3p, miR-148b-3p, and miR-148a-3p in H460, A549, H460/A549-derived LCSLCs, and BEP2D cells.*p < 0.05; **p < 0.01; ***p < 0.001

To investigate the potential transcriptional relationship between DNMT1, miR-152-3p, and SOS1, we initially employed TargetScan (https://www.targetscan.org/vert_80/) to predict the potential target genes of miR-152-3p. The analysis three family genes of miR-152-3p, including miR-152-3p, miR-148a-3p, and miR-148b-3p.The K_D_ values of miR-152-3p and DNMT1 were greater than those of miR-148a-3p and miR-148b-3p (Table 1A). Subsequently, we then assessed the expression levels of miRNAs in all cells using RT-qPCR. The results indicated that miR-152-3p exhibited lower expression levels in LCSLCs corresponding to A549 and H460 cells (Fig. 1C and 1D).Additionally, within the DNMT family, comprising DNMT1, DNMT3a, and DNMT3b [29], we determined the transcript levels of the DNMT family members in A549, H460 cell lines and their corresponding LCSLCs and BEP2D cell lines. The results indicated that DNMT1 exhibited higher levels in the corresponding LCSLCs (Fig. 1A and B).

It has been suggested that the miR-152/148 family may disrupt multiple signaling pathways in B lymphocytes through SOS1 and SOS2 [31]. Moreover, miR-148, in conjunction with SOS2, plays a role in the EMT phenotype of NSCLCs [38]. Subsequently, we used TargetScan to predict the correlation score of the miR-148/152 family with SOS1, which indicated that miR-152-3p has higher K_D_ values (Table 1B)*.* Consequently, we posited that miR-152/SOS1 may be related to LCSLCs. To further investigate this, we performed RT-qPCR to determine the transcript levels of SOS1 and SOS2, which revealed that SOS1 exhibited higher expression levels in A549 and H460 cells corresponding to LCSLCs (Fig. 1E and F).Therefore, miR-152-3p is negatively associated with DNMT1 and SOS1 in LCSLCs. To this end, we will conduct a series of studies explaining the mechanism by which DNMT, miR-152-3p, and SOS1 affect LCSLCs.

## DNMT1 and SOS1 were positively correlated, but miR-152-3p was negatively correlated with metastasis and progression of NSCLC

**Fig. 2:**
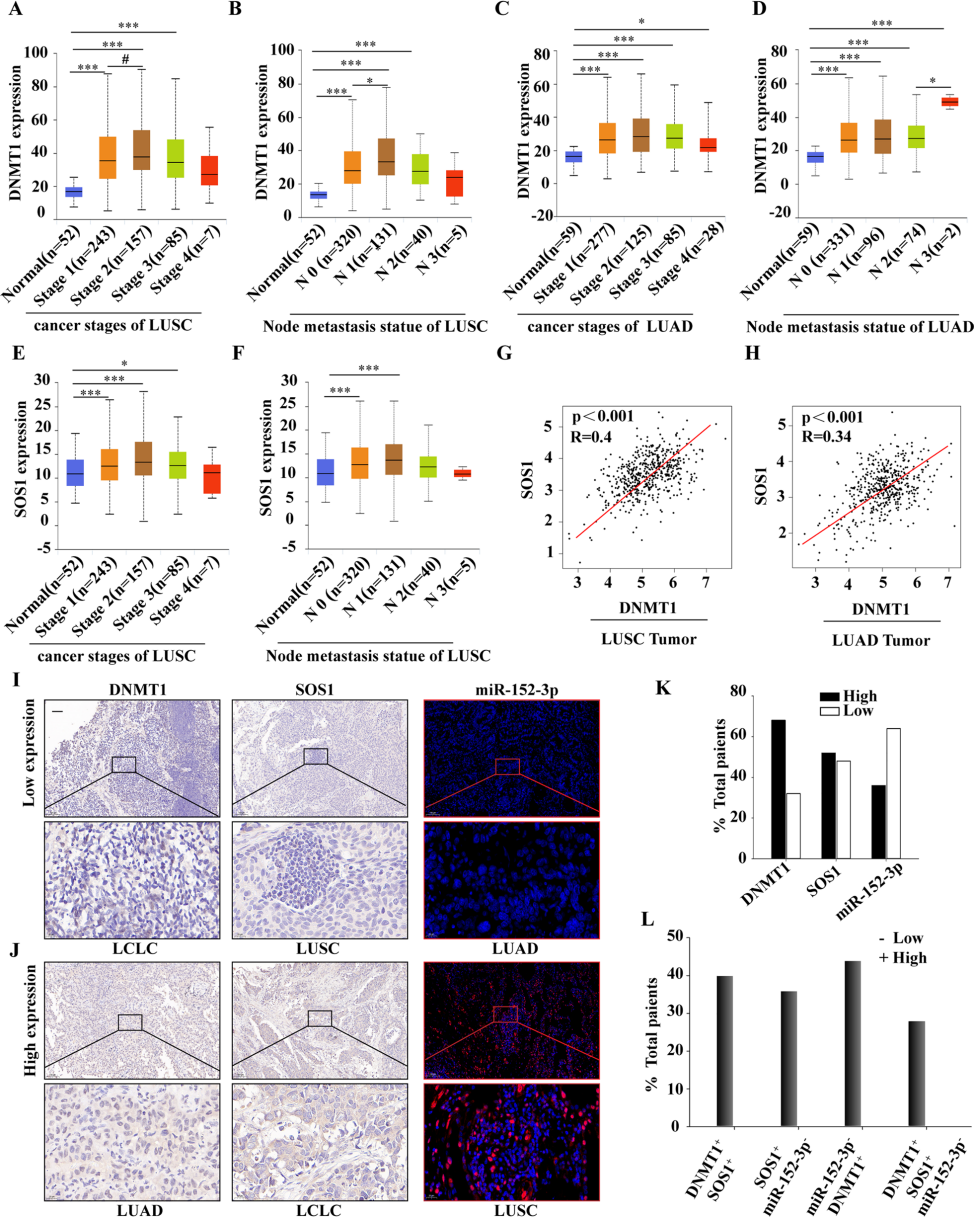
GEPIA database and tissue slice detection of DNMT1, SOS1, and miR-152-3p expression in NSCLCs **A** DNMT1 expression at each stage of LUSC; **B** DNMT1 expression at each metastatic stage of LUSC; **C** DNMT1 expression at each stage of LUAD; **D** DNMT1 expression at each metastatic stage of LUAD; **E** SOS1 expression in each metastatic stage of LUSC; **F** Expression levels of SOS1 in each metastatic stage of LUSC; **G** Correlation of DNMT1 and SOS1 expression in LUSC; **H** Association of DNMT1 and SOS1 expression in LUAD; **I** DNMT1, SOS1, and miR-152-3p low-expression representative images; **J** DNMT1, SOS1, and miR-152-3p high-expression representative images; **K** Percentage of high and low DNMT1, SOS1, and miR-152-3p expression in 25 NSCLC tumor sections; **L**The percentage of DNMT1 high-expression, and (or) SOS1 high-expression and (or) miR-152-3p low-expression in 25 non-small cell tumor sections.*p < 0.05; **p < 0.01; ***p < 0.001

In our analysis, we utilized the GEPIA database (http://gepia.cancer-pku.cn/) to investigate the expression patterns of DNMT1 and SOS1 in various stages and node metastasis in NSCLCs. The results revealed a significant association between DNMT1 expression and tumor progression and metastasis in LUSC and LUAD (Fig. 2A–D). Furthermore, SOS1 expression was found to be linked to tumor progression and metastasis in LUSC (Fig. 2E and F). Additionally, when we analyzed the correlation between DNMT1 and SOS1 in LUSC and LUAD, a positive correlation emerged, as depicted in Fig. 2G, H.These results suggest that the expression of DNMT1, miR-152-3p, and SOS1 may be associated with the metastasis and progression of NSCLC.

To verify whether there is differential expression of DNMT1, miR-152-3p, and SOS1 in the pathological tissues of NSCLC, we collected 25 pathological tissues from NSCLC patients. Immunohistochemical results revealed that DNMT1 and SOS1 were highly expressed in 68% and 52% of these tissues, respectively. Additionally, the results of the FISH assay indicated that miR-152-3p was downregulated in 64% of the samples (Fig. 2I and J).Both DNMT1 and SOS1 exhibited high expression, accompanied by a low expression (28%) of miR-152-3p (Fig. 2K).

## Comparison of self-renewal ability with tumor growth ability

**Fig. 3:**
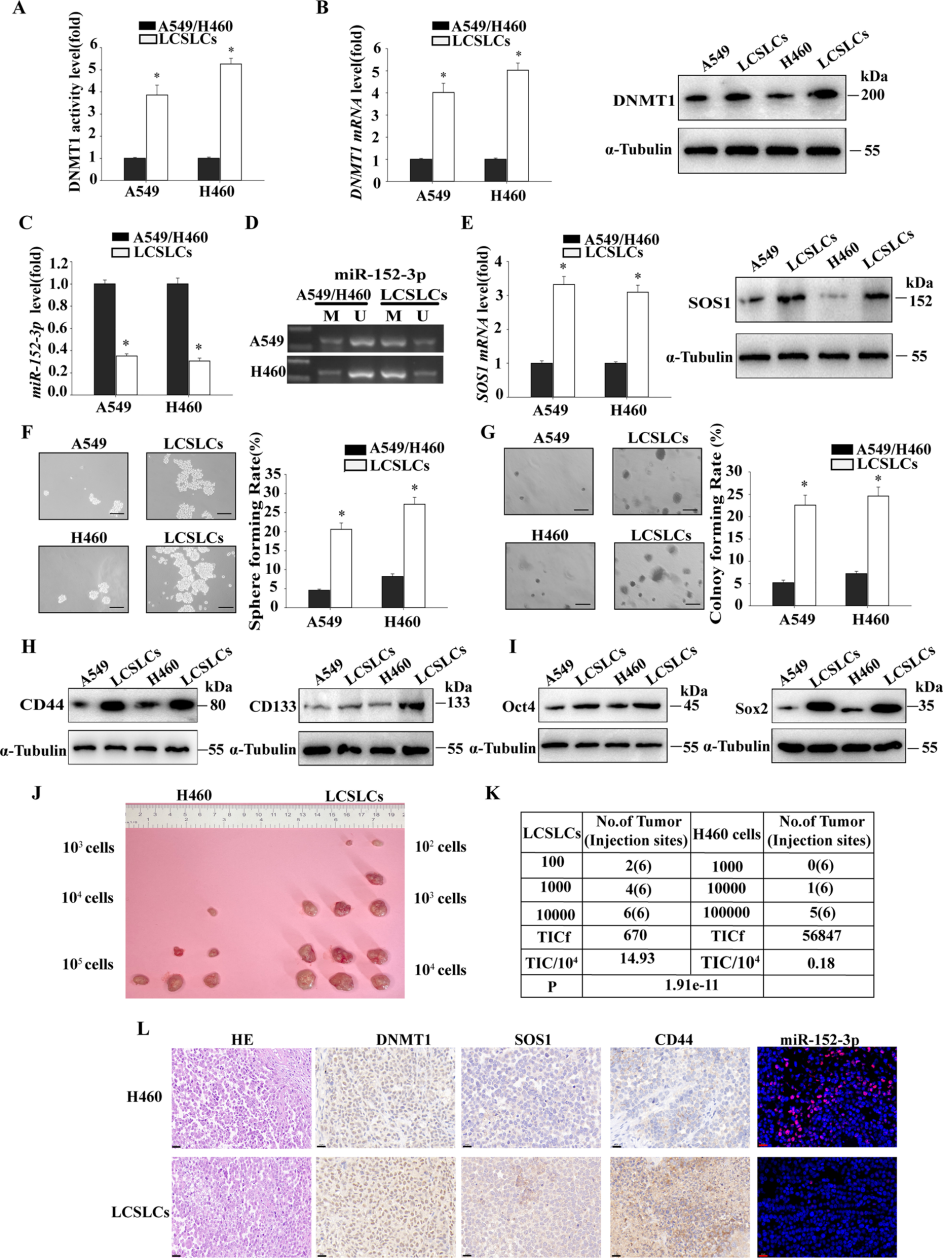
Comparison of self-renewal and tumor growth between H460 or A549 cells with the corresponding LCSLCs **A** DNMT1 activity; **B** DNMT1 mRNA and protein expression; **C** miR-152-3p levels; **D** miR-152-3p promoter methylation in H460 or A549 cells and their corresponding LCSLCs; **E** SOS1 mRNA and protein expression; **F**, **G** A549 and H460 cells exhibit similarities with their corresponding LCSLCs in terms of both sphere and colony formation rates (scale bar, 100 μm); **H**, **I** CD44, CD133, Oct4, and Sox2 protein expression. All experiments were conducted in triplicate. *p < 0.05 vs. A549 or H460 cells. The in vivo tumorigenicity of H460 tumorigenic cells and their LCSLCs **J**, including tumor-initiating cell frequency **K**, histology, DNMT1, SOS1, CD44, and miR-152-3p expression (scale bar, 50 μm) **L**. Data were obtained from xenografts at six vaccination sites (n = 6 graft tumors)

Several studies have demonstrated that DNMT1 expression is regulated by miR-152, and this regulatory mechanism is closely linked to angiogenesis, development, and metastasis in multiple tumors [39–41]. Additionally, miRNAs and SOS1 expression have been linked to radiation resistance, prognosis, and apoptosis in various tumors, serving as valuable detection biomarkers [42–44]. To delve deeper into the role of DNMT1/miR-152-3p/SOS1 expression in the self-renewal and tumor growth of NSCLCs, we conducted a comparative analysis. We compared the activity of DNMT1 in the H460 and A549 cells with their corresponding LCSLCs.The results revealed higher DNMT1 activity in the corresponding LCSLCs (Fig. 3A). Furthermore, LCSLCs exhibited higher DNMT1 mRNA and protein levels (Fig. 3B). In addition, miR-152-3p level was lower in LCSLCs compared to H460 and A549 cells (Fig. 3C). Notably, MSP experiments demonstrated a significant increase in the methylation level of the miR-152-3p promoter in LCSLCs (Fig. 3D). Moreover, LCSLCs exhibited higher SOS1 mRNA and protein expression levels (Fig. 3E).

We also assessed the self-renewal potential and colony-forming ability, which were found to be significantly higher in LCSLCs (Fig. 3F and G). Numerous studies have identified CD133 and CD44 as common lung CSC markers [45], as well as the upregulation of stem cell-associated transcription factors Oct4 and Sox2 in CSCs [10, 11]. In this study, we evaluated the expression levels of CD133, CD44, Oct4, and Sox2 in A549 and H460 cells as well as the derived LCSLCs. The results indicated that the expression of all these stem cell-associated genes were higher in LCSLCs than in H460 and A549 cells (Fig. 3H and I).

To assess the tumorigenicity of LCSLCs in vivo, we conducted subcutaneous xenograft assays in nude mice using H460-derived LCSLCs. The results indicated that H460-derived LCSLCs were more tumorigenic than H460 cells in vivo (Fig. 3J and K). Moreover, HE staining, immunohistochemistry, and in FISH (Fig. 3L) revealed that the level of DNMT1, SOS1, miR-152-3p and CD44 in H460-derived LCSLCs were higher than those in H460 cells, while the level of miR-152-3p was the opposite. Our results indicated that LCSLCs derived from the A549 and H460 cells exhibited stronger self-renewal and tumor growth abilities, compared with the A549 and H460 cells.Furthermore, H460 cell-derived spheroids display greater tumorigenicity in vivo, likely attributable to high levels of DNMT1, low levels of miR-152-3p, miR-152-3p promoter hypermethylation, and SOS1 overexpression.

## DNMT1 plays a vital role in maintaining the self-renewal and tumor growth of NSCLC

**Fig. 4:**
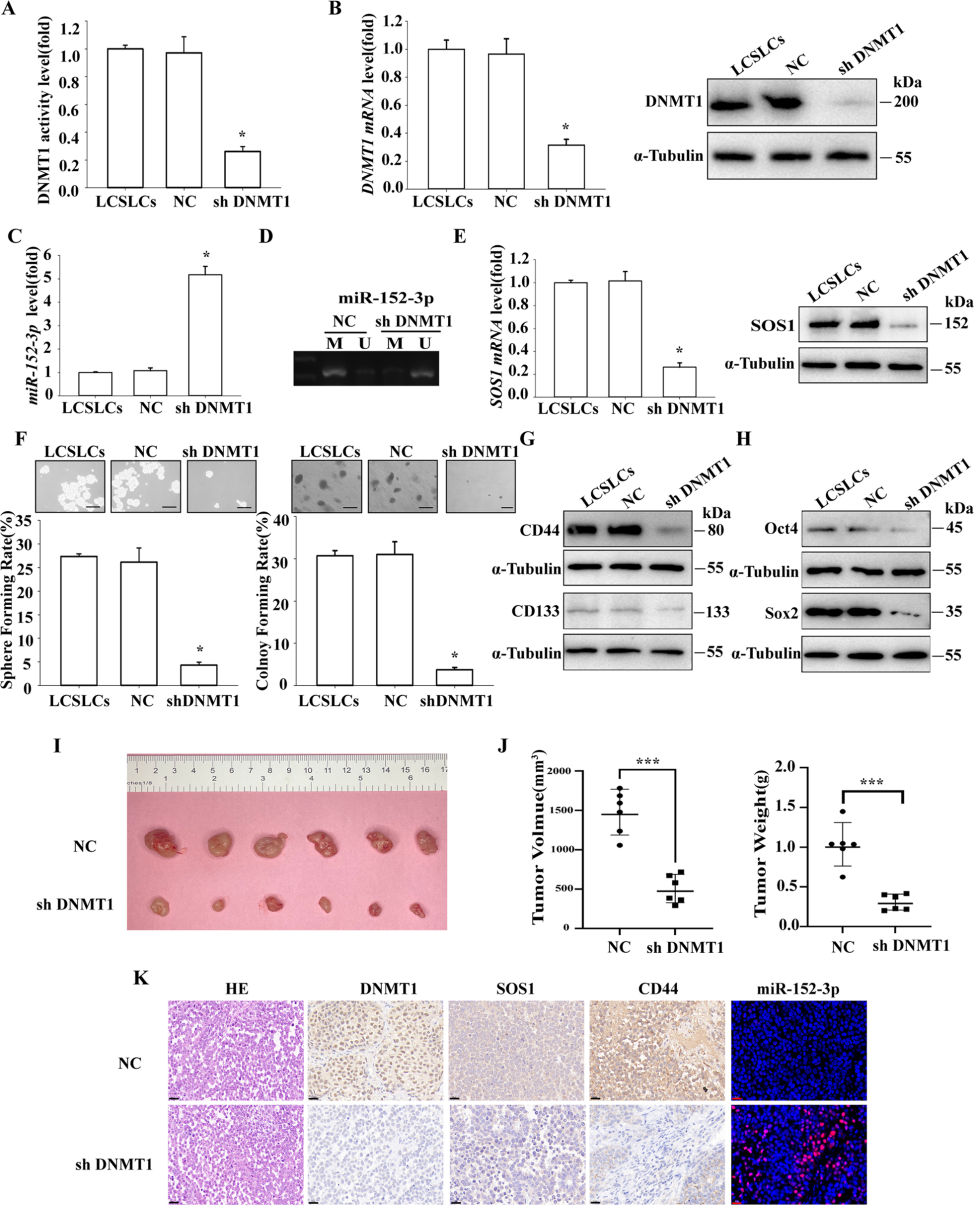
Effect of shDNMT1 on the self-renewal and tumor growth of H460-derived LCSLCs **A** DNMT1 activity; **B** DNMT1 mRNA and protein expression; **C** miR-152-3p levels; **D** miR-152-3p promoter methylation; **E** SOS1 mRNA and protein expression; **F** Rates of sphere and colony formation (scale bar, 100 μm); **G**, **H** Protein expression of CD44, CD133, Oct4, and Sox2. All experiments were conducted in triplicate. *p < 0.05 vs. NC; **I** Subcutaneous xenografts of LCSLCs (1 × 10^5^) treated with shNC or shDNMT1; **J** Tumor volume and weight; **K** H&E staining and immunohistochemistry for the expression of DNMT1, SOS1, and CD44 proteins, and in situ immunofluorescence hybridization of miR-152-3p (scale bar, 50 μm). Data were obtained from xenografts from six mice per group. *p < 0.05 vs. NC

To investigate the role of DNMT1 in LCSCLs, we used shDNMT1 to knock down DNMT1 expression levels in LCSLCs. Protein activity assays revealed a significant reduction in DNMT1 activity levels (Fig. 4A), as well as a decrease in DNMT1 mRNA and protein levels (Fig. 4B). Furthermore, the level of miR-152-3p was up-regulated following sh DNMT1 treatment (Fig. 4C). Notably, there was a decrease in miR-152-3p promoter methylation (Fig. 4D), as well as reduced SOS1 mRNA and protein level (Fig. 4E). Additionally, DNMT1 treatment resulted in diminished sphere formation and colony formation abilities (Fig. 4F), along with reduced expression levels of CD44, CD133 (Fig. 4G), Oct4, and Sox 2 (Fig. 4H). In our nude mouse graft model, the knockdown of DNMT1 led to the inhibition of tumor growth (Fig. 4I and J), accompanied by decreased protein expression of DNMT1, SOS1, and CD44 and increased levels of miR-152-3p (Fig. 4K). These findings suggest that the knockdown of DNMT1 suppressed the self-renewal and tumor growth of H460-derived LCSLCs, possibly by upregulating miR-152-3p due to promoter hypomethylation.

In a parallel experiment, we utilized DNMT1-cDNA to induce the overexpression of DNMT1 levels in H460 cells. The results showed that following treatment with DNMT1-cDNA, there was a notable increase in DNMT1 activity (Additional file 1: Fig. S1A) and a significant increase in mRNA (Additional file 1: Fig. S1B) and protein (Additional file 1: Fig. S1C) expression levels. Additionally, miR-152-3p levels exhibited a downregulation (Additional file 1: Fig. S1D), while SOS1 mRNA and protein levels showed an upregulation. Importantly, sphere and colony formation were significantly enhanced (Additional file 1: Fig. S1E and F), along with increased expression levels of CD44, CD133 (Additional file 1: Fig. S1G), Oct4, and Sox2 (Additional file 1: Fig. S1H). These results further emphasize the crucial role of DNMT1 in maintaining self-renewal and tumor growth of CSLC in H460 cells.

## miR-152-3p regulates DNMT1 expression and CSLC self-renewal and tumor growth of NSCLC cells

**Fig. 5:**
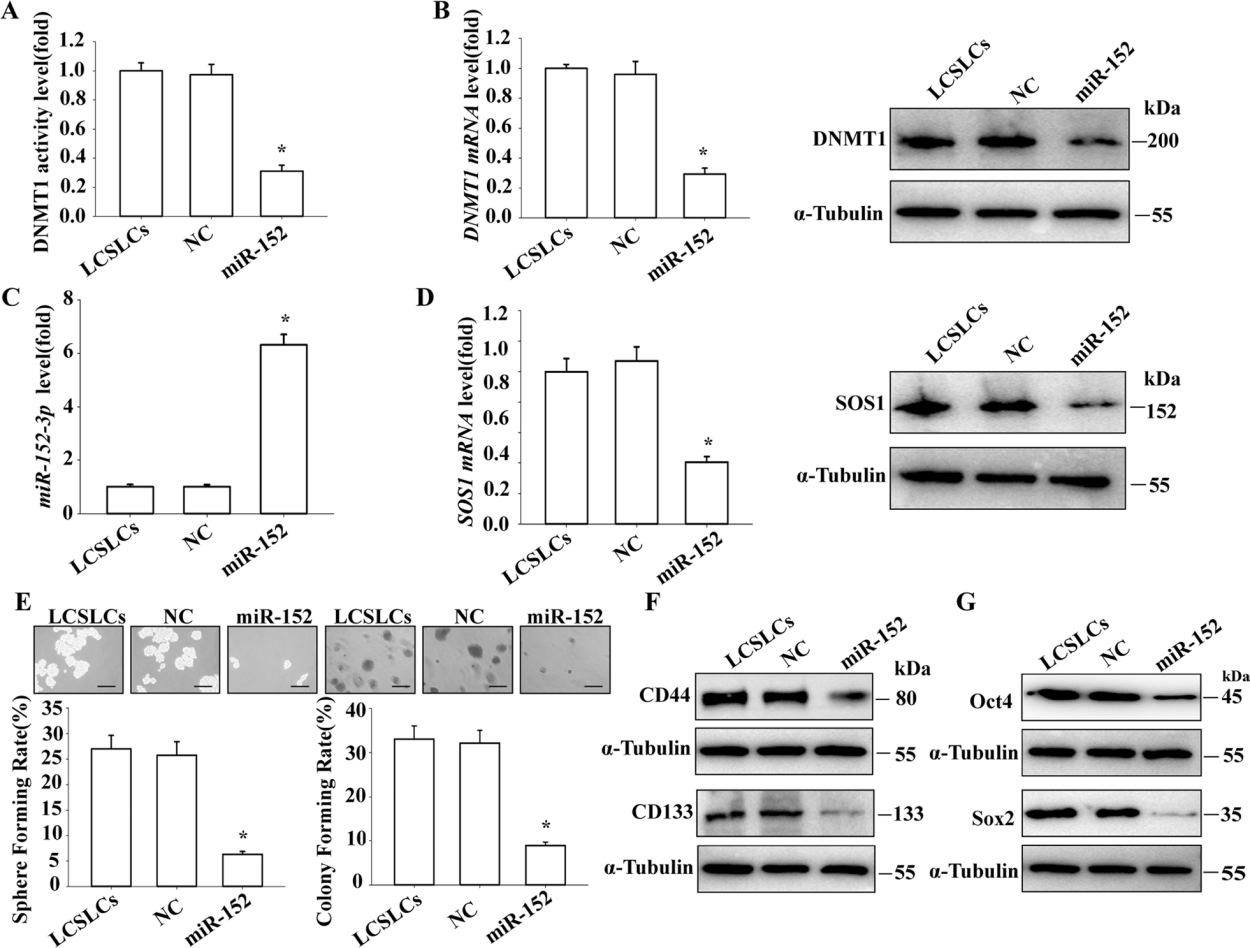
Effect of miR-152-3p mimic on the self-renewal and tumor growth of H460-derived LCSLCs **A** DNMT1 activity; **B** DNMT1 mRNA and protein levels; **C** miR-152-3p levels; **D** SOS1 mRNA and protein levels; **E** Rates of sphere and colony formation (scale bar, 100 μm); **F** Expression levels of CD44 and CD133; **H** Expression levels of Oct4 and Sox2. All experiments were conducted in triplicate. *p < 0.05 vs. NC

To investigate the relationship between DNMT1 and miR-152-3p, LCSLCs were treated with either miR-NC or miR-152-3p mimics. The results showed that after miR-152-3p mimic treatment, DNMT1 activity (Fig. 5A), mRNA (Fig. 5B), and protein (Fig. 5C) levels in LCSLCs were significantly reduced compared to the miR-NC treatment group. In addition, the mRNA and protein levels of SOS1 were downregulated (Fig. 5D), and both sphere and colony formation capabilities were reduced (Fig. 5E). Levels of CD44, CD133, Oct4, and Sox2 (Fig. 5F and G) were also reduced.

In addition, we used a miR-152-3p inhibitor to knock down miR-152-3p levels in H460 cells. The results showed that after miR-152-3p inhibitor treatment, DNMT1 activity (Additional file 1: Fig. S2A) as well as mRNA (Additional file 1: Fig. S2B) and protein levels were significantly upregulated, while miR-152-3p levels were downregulated (Additional file 1: Fig. S2C). Additionally, SOS1 mRNA (Additional file 1: Fig. S2D) and protein expression levels were upregulated. Notably, sphere formation and colony formation capacity were significantly enhanced (Additional file 1: Fig. S2E and F), and the levels of CD44, CD133 (Additional file 1: Fig. S2G), Oct4, and Sox2 (Additional file 1: Fig. S2H) were upregulated. These results further support the notion that DNMT1/miR-152-3p plays a role in maintaining the self-renewal and tumor growth of H460 cells through negative regulation.

## SOS1 influences the CSLC features of LCSLCs without impacting DNMT1 function or miR-152-3p levels

**Fig. 6:**
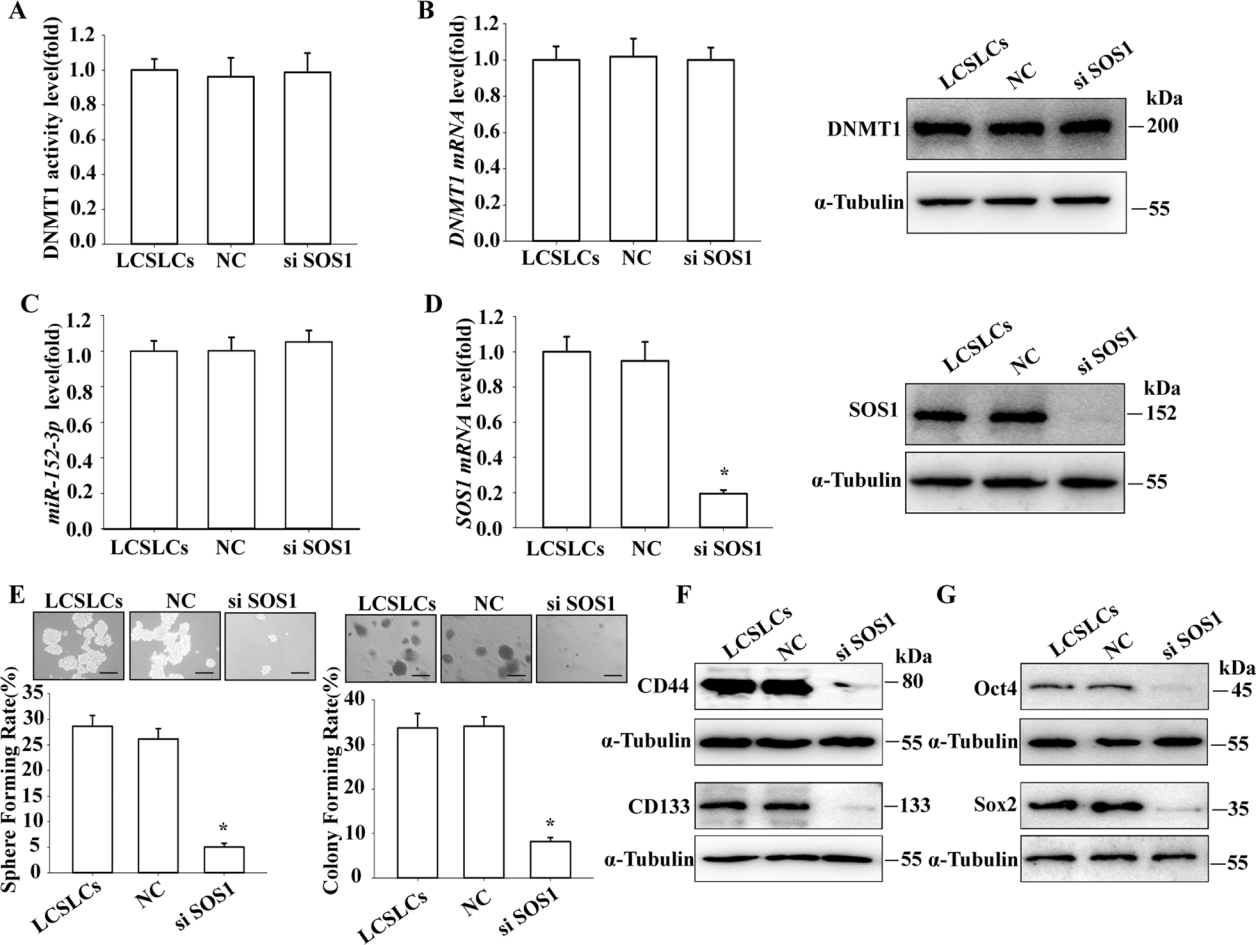
Effect of siSOS1 on the CSLC characteristics of H460-derived LCSLCs **A** DNMT1 activity; **B** DNMT1 mRNA and protein expression; **C** miR-152-3p levels; **D** SOS1 mRNA and protein levels; **E** Rates of sphere and colony formation (scale bar, 100 μm); **F**, **G** Expression levels of CD44, CD133, Oct4, and Sox2. All experiments were conducted in triplicate. *p < 0.05 vs. NC

To investigate the relationship between SOS1 and DNMT1/miR-152-3p and their impact on the self-renewal and tumor growth of LCSLCs, LCSLCs were treated with siSOS1. The results showed that DNMT1 activity (Fig. 6A), as well as mRNA and protein level in H460-derived LCSLCs, remained unaltered (Fig. 6B), and there was no significant effect on miR-152-3p expression when compared to H460 cells (Fig. 6C). However, the mRNA and protein level of SOS1 were significantly decreased (Fig. 6D). Importantly, siSOS1 treatment reduced the sphere and colony formation capacities of LCSLCs (Fig. 6E). Western blot experiments also showed that siSOS1 downregulated the expression levels of CD44, CD133 (Fig. 6F), Oct4, and Sox 2 (Fig. 6G).

To further elucidate the mechanism of DNMT1/miR-152-3p/SOS1 in NSCLC, we overexpressed SOS1 in H460 cells using SOS1-cDNA. The results showed that after SOS1-cDNA treatment, the DNMT1 activity of H460 cells (Additional file 1: Fig. S3A), as well as DNMT1 mRNA (Additional file 1: Fig. S3B) and protein, levels remained unaltered. miR-152-3p level were increased (Additional file 1: Fig. S1C). However, the SOS1 mRNA (Additional file 1: Fig. S3D) and protein level were increased. Importantly, sphere and colony formation capabilities were significantly increased (Additional file 1: Figs. S3E and F), as well as the level of CD44, CD133 (Fig. S3G), Oct4, and Sox2 (Additional file 1: Fig. S3H). These results further underscore the role of DNMT1/miR-152-3p negatively regulating SOS1 in promoting the stem-like self-renewal and tumor growth of H460 cells.

## DNMT1 and SOS1 as targets of miR-152-3p in H460-derived LCSLCs

**Fig. 7:**
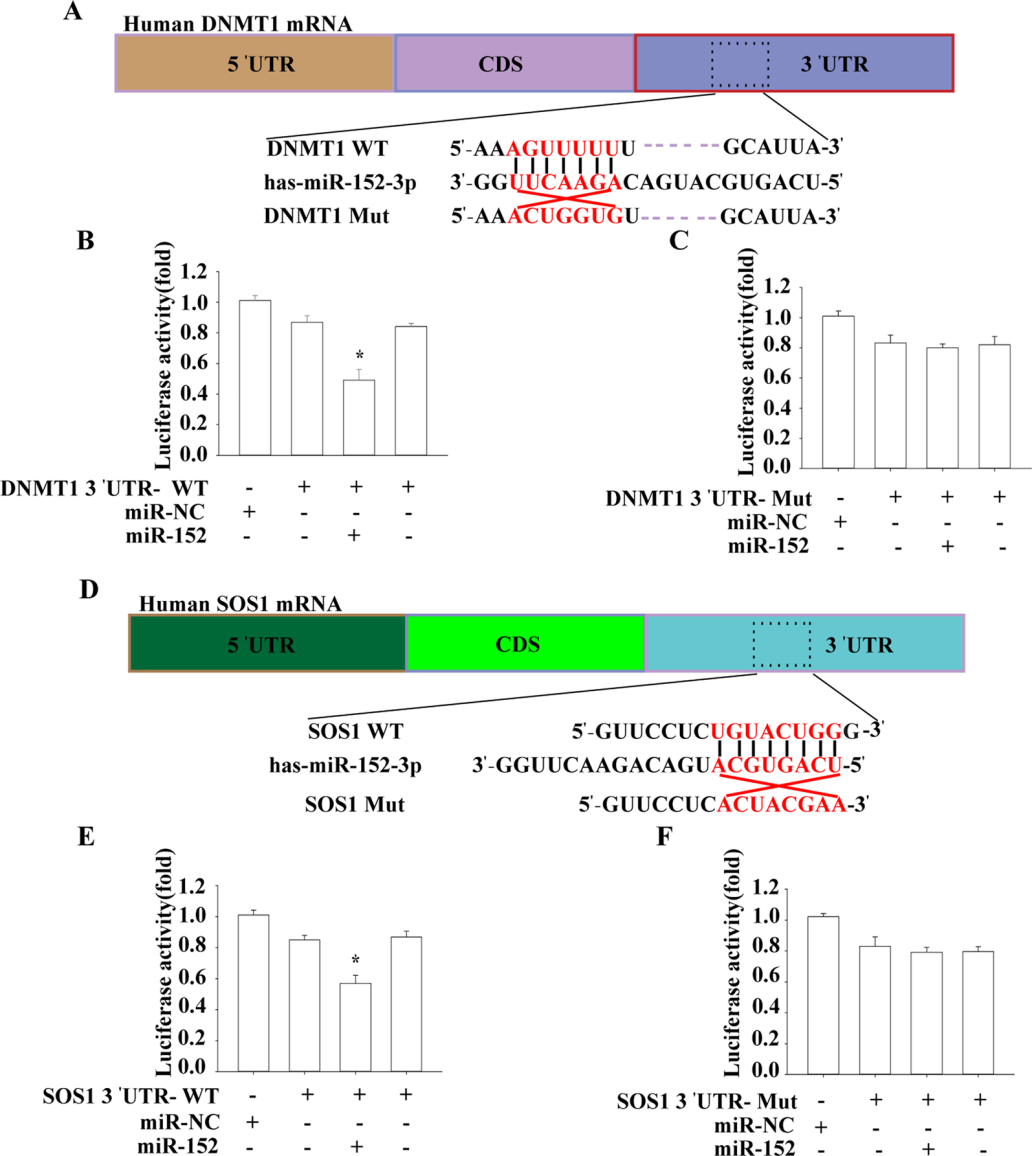
DNMT1 and SOS1 as direct targets of miR-152-3p in the H460-derived LCSLCs **A** Schematic representation of the binding sites of miR-152-3p and DNMT1 3'UTR of wild-type (wt) and mutant (mut) clones cloned into the luciferase reporter arrangement of pLUC control; **B** and **C** Relative luciferase activity in H460-derived LCSLCs was determined after co-transfection of the DNMT1 3'UTR wild-type or mutant plasmid with a *miR-152-3p* mimic or negative control. **D** Schematic representation of the binding sites of miR-152-3p and SOS1 3'UTR of wild-type (wt) and mutant (mut) clones cloned into the luciferase reporter arrangement of the pLUC control; **E** and **F** Relative luciferase activity in H460-derived LCSLCs was determined after co-transfection of the SOS1 3'UTR wild-type or mutant plasmid with a miR-152-3p mimic or negative control. *p < 0.05 vs. NC

To determine whether DNMT1 is a direct target of miR-152-3p, we performed luciferase reporter in H460-derived LCSLCs. We aimed to find the precise binding site of miR-152-3p to the 3'-UTR of DNMT1 mRNA. The luciferase reporter assay revealed a significant inhibition in luciferase activity when miR-152-3p-mimics were co-transfected with DNMT1-3'-UTR-wt (Fig. 7B and C). However, this effect was not observed when miR-152-3p mimics were co-transfected with DNMT1-3'-UTR-Mut (Fig. 7B and C). These results suggest that DNMT1 is a distinct target of miR-152-3p in the H460-derived LCSLCs.

Several studies have demonstrated the relationship between SOS1 and miRNAs in the context of tumor radiation resistance, prognosis, and apoptosis [42–44]. In our luciferase reporter assays, we observed a significant reduction in luciferase activity when miR-152-3p mimics were co-transfected with SOS1-3'-UTR-wt (Fig. 7E and F). However, this effect was not observed when miR-152-3p mimics were co-transfected with SOS1-3'-UTR-Mut (Fig. 7E and F). Thus, the cumulative evidence presented in this study supports the hypothesis that the constitutive activation of DNMT1 leads to the silencing of miR-152-3p through promoter methylation. The low level of miR-152-3p, in turn, activates DNMT1 by relieving its inhibition.

## The DNMT1/miR-152-3p/SOS1 axis also regulates self-renewal and tumor growth in A549-derived LCSLCs

**Fig. 8:**
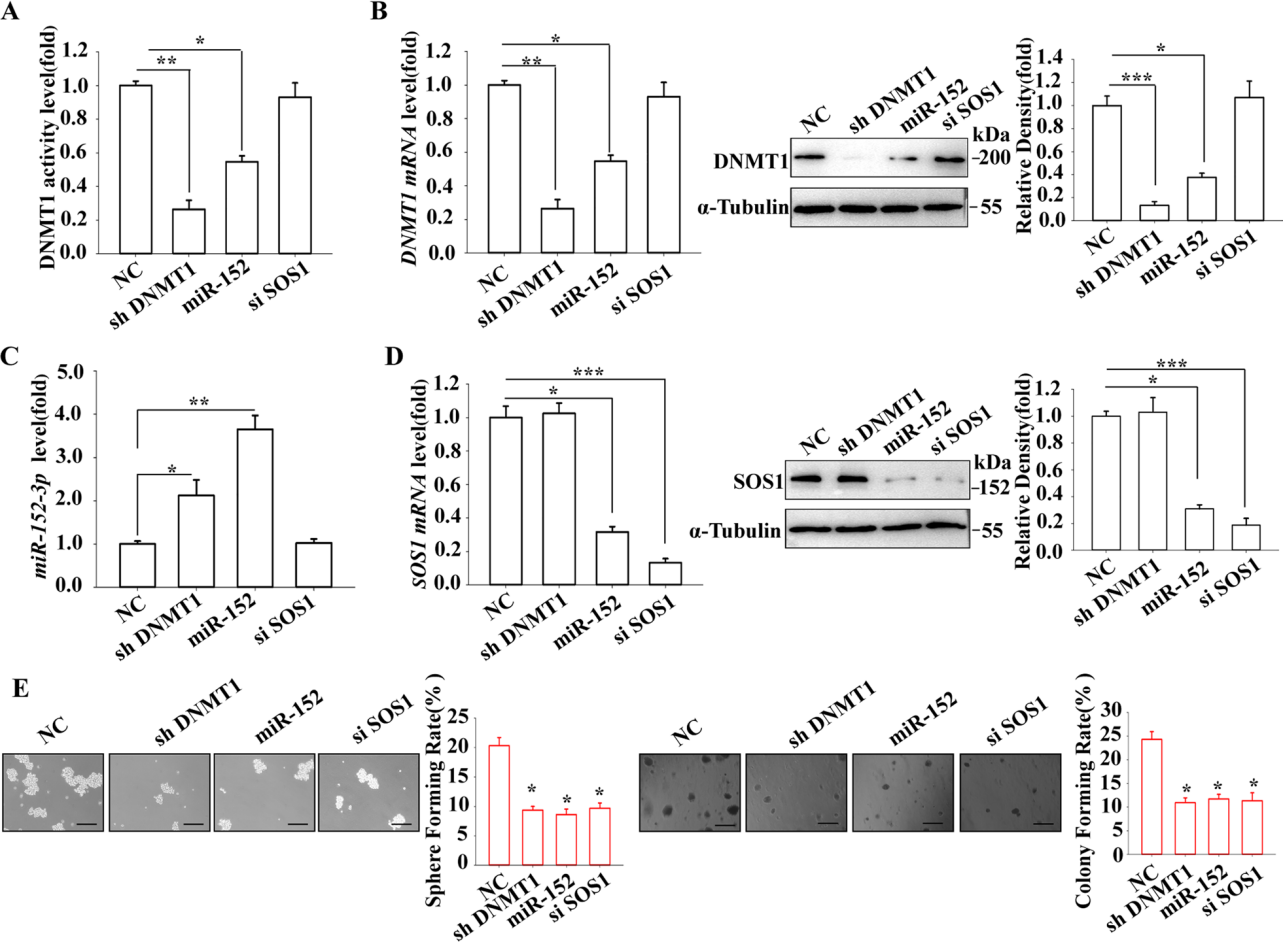
Effects of shDNMT1, miR-152-3p mimic, and siSOS1 on self-renewal and tumor growth of A549-derived LCSLCs DNMT1 **A** DNMT1 activity; **B** DNMT1 mRNA and protein levels; **C** miR-152-3p levels; **D** SOS1 mRNA and protein levels; **E** Rates of sphere and colony formation (scale bar, 100 μm). All experiments were conducted in triplicate. *p < 0.05; ** *p* < 0.01; ***p < 0.001 vs. NC

**Fig. 9:**
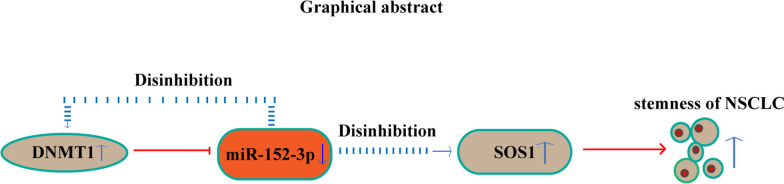
The graphical summary shows the specific mechanism of the DNMT1/miR-152-3p/SOS1 signaling axis in maintaining the self-renewal and tumor growth in NSCLC cells

To further elucidate the mechanism of action of the DNMT1/miR-152-3p/SOS1 axis in the NSCLC, A549-derived LCSLCs were treated with shDNMT1, miR-152-3p-mimic, and siSOS1 to assess their effects on DNMT1, miR-152-3p, and SOS1 levels, as well as CSLC features in A549-derived LCSLCs. Comparing the NC group with the respective treatment groups revealed that shDNMT1 and miR-152 mimic decreased DNMT1 activity (Fig. 8A), mRNA and protein expression (Fig. 8B), and increased miR-152-3p levels (Fig. 8C), but these changes were not observed in the siSOS1 treatment group. However, siSOS1 decreased the mRNA and protein levels of SOS1 in the A549-derived LCSLCs (Fig. 8D). Importantly, shDNMT1, miR-152-3p-mimic, and siSOS1 significantly reduced the spheroids and colony formation capacities in A549-derived LCSLCs (Fig. 6E). The results indicate that the DNMT1/miR-152-3p pathway promotes the self-renewal and tumor growth of A549-derived LCSLCs through the regulation of SOS1 expression (Fig. 9).

## Discussion

We aimed to investigate the relationship between DNMT1, miR-152-3p, and SOS1, which collectively regulate self-renewal and tumor growth in NSCLC cells. To the best of our knowledge, this is the first report highlighting the antagonistic relationship between DNMT1 and miR-152-3p, which enhances the self-renewal and tumor growth of NSCLC cells in vivo. This is followed by the subsequent upregulation of SOS1 levels upon miR-152-3p silencing. The pathological results suggested that some patients exhibited high expression of DNMT1 and SOS1, accompanied by low expression of miR-152-3p. Our report confirms the possibility of DNMT1/miR-152-3p/SOS1 regulating the tumor growth and self-renewal of LCSLCs, providing new insights into the initiation and progression of NSCLC.

In recent years, there has been a growing focus on the epigenetic regulation of DNMT1 [45] and miR-152-3p in premalignant cells [39, 41]. DNMT1 exhibits unusual activation in tumors and CSCs, playing a pivotal role in mediating the methylation of downstream molecules, thereby inducing self-renewal and tumor growth of CSC [45]. In addition, DNMT1 is crucial for the maintenance of CSLC identity [46]. Our analysis of collected lung cancer specimens revealed elevated expression levels of DNMT1, CD44, and SOS1 in certain NSCLC tissues, establishing a positive correlation between DNMT1 and SOS1 in the progression of NSCLC. We hypothesize that DNMT1 activates SOS1 through miR-152-3p in epigenetic regulatory processes, with miR-152-3p expression affecting both DNMT1 and SOS1. This scenario appears to be highly relevant to cancer progression, particularly in promoting LCSLC features.

There is a mounting body of evidence suggesting that miRNA regulation via DNMT1 is intricately linked to aberrant mechanisms of promoter hypermethylation in tumors [26–28]. In particular, miR-152-3p has been identified as a tumor suppressor that is consistently downregulated in various cancer types [41–44]. Our study offers compelling evidence that reciprocal negative interaction between DNMT1 and miR-152-3p stimulates the emergence of LCSLC features and carcinogenicity. Knocking down DNMT1 led to an increase in miR-152-3p levels while concurrently decreasing LCSLC self-renewing and carcinogenicity. The introduction of miR-152-3p mimics inhibited both the transcriptional and post-transcriptional expression of DNMT1. Notably, neither the overexpression nor the knockdown of SOS1 had any discernible impact on the expression levels of DNMT1/miR-152-3p.

Furthermore, luciferase assays revealed that DNMT1 is a direct target of miR-152-3p, underscoring the specific relevance of miR-152-3p in regulating DNMT1 expression. These findings suggest that a reciprocally antagonistic modulation between DNMT1 and miR-152-3p could serve as a prospective therapeutic approach for NSCLC treatment through LCSLC targeting. This approach may also pave the way for the exploration of demethylating agents in the field of epigenetic cancer therapy.

SOS1 is a well-known oncogenic transcriptional factor implicated in the initiation, progression, and metastasis of multiple tumors [47, 48]. Development of inhibitors targeting SOS1 as potential therapeutic agents in cancer research [49–51]. However, the upstream regulatory mechanisms governing SOS1 connectivity in LCSLCs warrant further investigation. Our study aligns with this hypothesis, as we have observed that the si-SOS1 could replicate the inhibitory effects of miR-152-3p on self-renewal and tumor growth of LCSLC. Remarkably, increased expression of SOS1 reversed the inhibitory effects of miR-152-3p on self-renewing and carcinogenicity in LCSLCs, yet it did not exert any discernible impact on the expression of DNMT1 and miR-152-3p in LCSLCs.

Emerging evidence has already unveiled a link between DNMT1 and miR-152-3p in previous studies [29, 52]. However, the intricate mechanisms underlying the interplay between miR-152-3p and SOS1 in tumors remained largely unexplored. In this study, luciferase reporter assays were conducted in H460-derived LCSLCs to investigate the relationship between miR-152-3p and the SOS1 mRNA 3'UTR. These results provide a clear indication that SOS1 promotes self-renewal and tumor growth of LCSLC through a reciprocal negative regulation mechanism involving DNMT1 and miR-152-3p in NSCLC. Furthermore, miR-152-3p may upregulate SOS1 by activating DNMT1, thereby promoting self-renewal and tumor growth in LCSLCs.

This study establishes a critical role for the complex interplay between miR-152-3p, DNMT1, and SOS1 in regulating and maintaining self-renewal and tumor growth within LCSCLs. Furthermore, they provide insights into an epigenetic process with a potential for clinical translation in LCSCLs.

### Supplementary Information

**Additional file 1.** Supplementary materials include supplemental figures 1–3.

**Additional file 2.** Supplementary materials include supplemental tables 1–4.

## Data Availability

The datasets used and/or analysed during the current study are available from the corresponding author on reasonable request.

## Abbreviations

BSA: Bovine serum albumin
CSCLC: Cancer stem cell-like cell
CSC: Cancer stem cell
CD44: Cluster of differentiation-44
CD133: Cluster of differentiation-133
DMEM: Dulbecco’s modified Eagle’s medium
DMSO: Dimethyl sulfoxide
NSCLC: Non–small cell lung carcinoma
NANOG: Nanog homeobox
Oct4: Octamer-binding transcriptional factor 4
PBS: Phosphate buffered solution
DNMT1: DNA methyltransferase 1
SOS1: Son of sevenless homolog 1
TCGA: The Cancer Genome Atlas
NSCLC: Non-small cell lung cancer
