# Supplementary material for: DNMT1/miR-152-3p/SOS1 signaling axis promotes self-renewal and tumor growth of cancer stem-like cells derived from non-small cell lung cancer
Source: Clin Epigenetics. 2024 Apr 15;16:55. doi: 10.1186/s13148-024-01663-5 (PMC11020669; doi:10.1186/s13148-024-01663-5)
Supplement: Supplementary file 1 — Additional file 1. Supplementary materials include supplemental figures 1–3. [file 13148_2024_1663_MOESM1_ESM.docx]

**
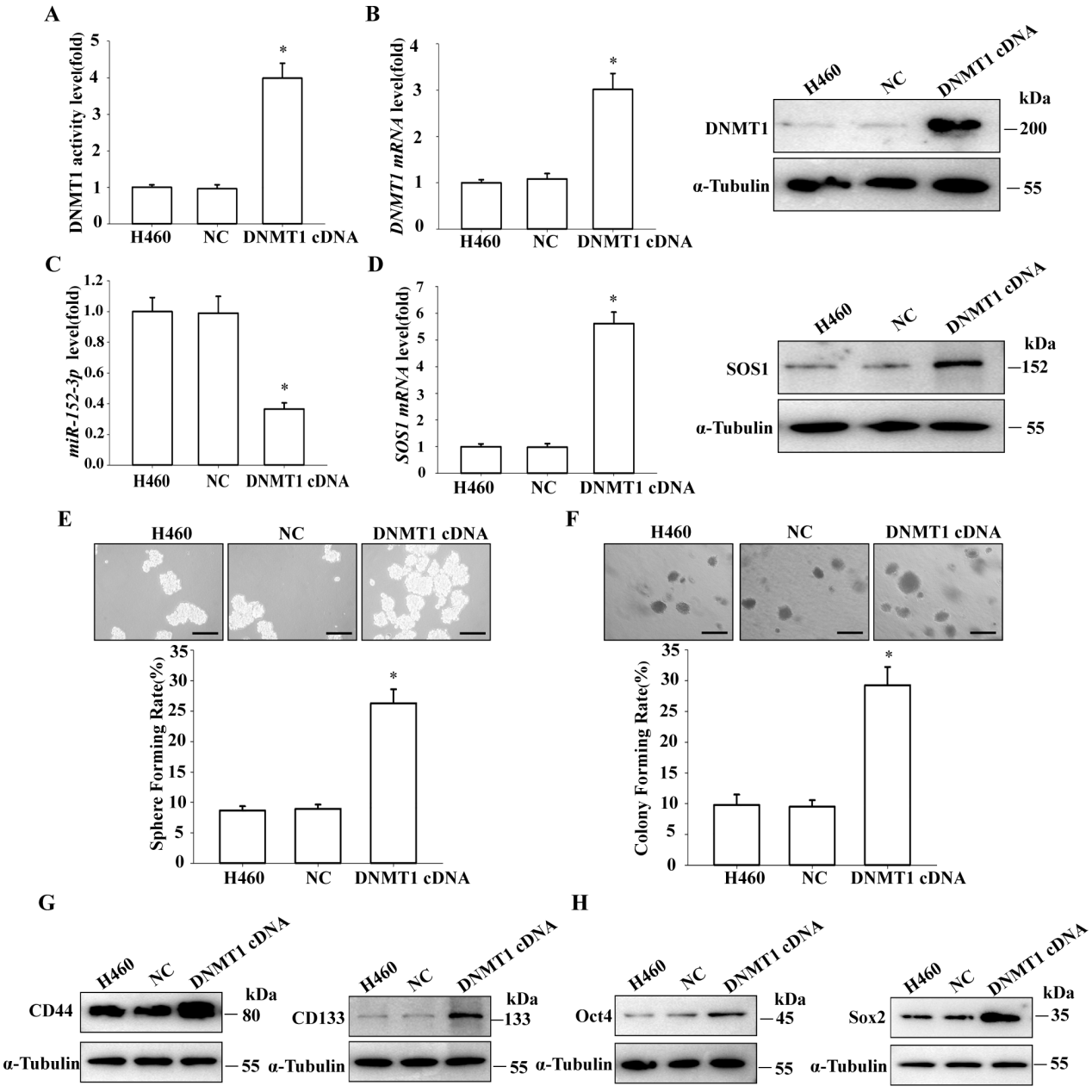
Fig S1 Effect of the DNMT1 cDNA on the CSLC properties of H460 cells**

(A)DNMT1 activity, (B)DNMT1 mRNA and protein levels, (C)miR-152-3p levels (D)SOS1 mRNA and protein levels.(E-F)Rate of formation and colony formation(scale bars, 100 μ m).(G) CD44, CD133, and (H)the protein levels of Oct4 and Sox2. The above data are all obtained from three repeated independent experiments, showing the results of one representative experiment * P <0.05 vs NC.


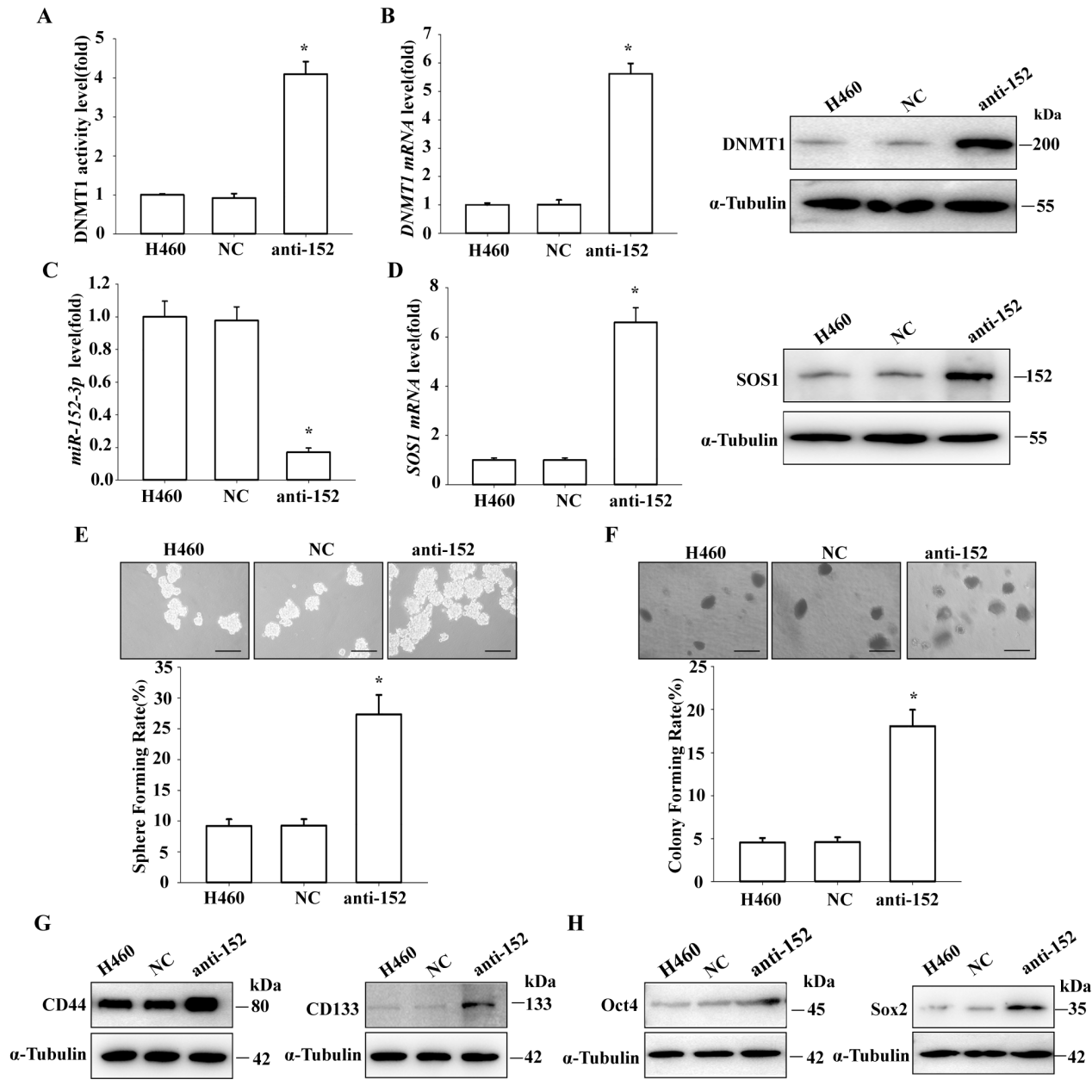


**Fig S2 Effect of the miR-152-3p inhibitor on the CSLC properties of H460 cells**

1. DNMT 1 activity,(B)DNMT1 mRNA and protein levels, (C) miR-152-3p levels.(D)SOS1 mRNA and protein levels.(E-F)Rate of formation and colony formation(scale bars, 100 μ m).(G) CD44, CD133, and (H) the protein levels of Oct4 and Sox2. The above data are all obtained from three repeated independent experiments, showing the results of one representative experiment.* P <0.05 vs NC.

*
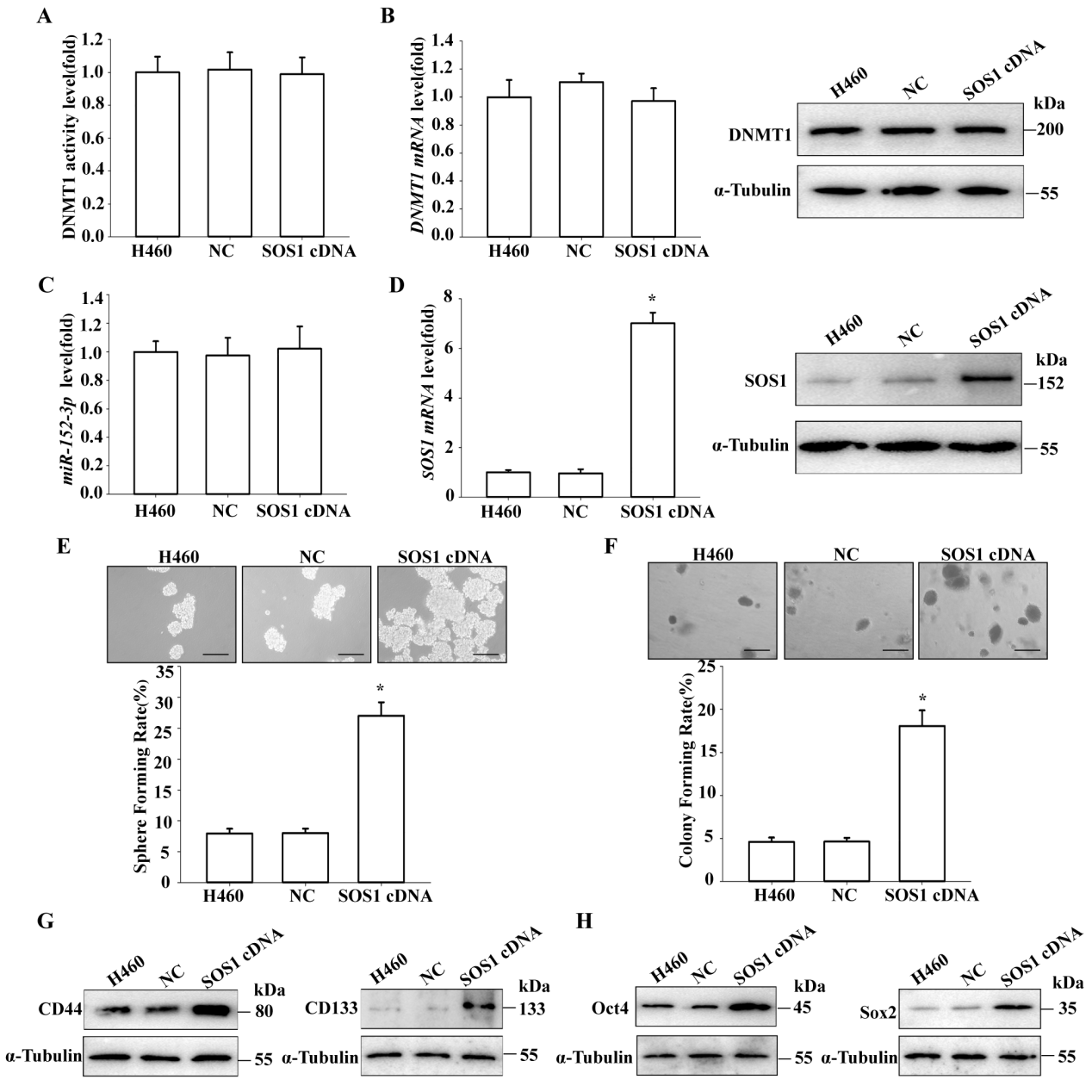
*

**Fig S3 Effect of SOS1 cDNA on the CSLC properties of H460 cells**

(A)DNMT1 activity, (B)DNMT1 mRNA and protein levels, (C) *miR-152-3p* levels.(D)SOS1 mRNA and protein levels.(E-F)Rate of formation and colony formation(scale bars, 100μm).(G) CD44, CD133, and (H) the protein levels of Oct4 and Sox2. The above data are all obtained from three repeated independent experiments, showing the results of one representative experiment * P <0.05 vs NC.
