# Supplementary material for: DNMT1/miR-152-3p/SOS1 signaling axis promotes self-renewal and tumor growth of cancer stem-like cells derived from non-small cell lung cancer
Source: Clin Epigenetics. 2024 Apr 15;16:55. doi: 10.1186/s13148-024-01663-5 (PMC11020669; doi:10.1186/s13148-024-01663-5)
Supplement: Supplementary file 2 — Additional file 2. Supplementary materials include supplemental tables 1–4. [file 13148_2024_1663_MOESM2_ESM.doc]

**Table S1**

Primers F/R Sequence（5ʹ-3ʹ） Product length

Accenssion (NM_001130823)

DNMT1 F CCATCAGGCATTCTACCA 132

DNMT1 R CGTTCTCCTTGTCTTCTCT

Accenssion (NM_001320893 XM_011532665)

DNMT3a F GGCAGTGGCTGAGAAGAAA 200

DNMT3a R CTGCTTTGGTGGCATTCTTG

Accenssion (NM_001207056)

DNMT3b F GGCCCAAGTAAACCTAGCTC 157

DNMT3b R CTCCCTTCATGCTTTCCTGC

Accenssion (NM_001382394 XM_011533062)

SOS1 F GAGTGAATCTGCATGTCGGTT 177

SOS1 R CTCTCATGT TTGGCTCCTACAC

Accenssion (NM_006939 XM_043720)

SOS2 F CCGCAGCCTTACGAGTTCTTC  95

SOS2 R GGATGCACTTGTTCCTGAACC

Accenssion (NM_001256799)

GAPDH F GGAGCGAGATCCCTCCAAAAT 197

GAPDH R GGCTGTTGTCATACTTCTCATGG

Accenssion ([MI0000462](http://www.mirbase.org/cgi-bin/mirna_entry.pl?acc=MI0000462))

miR-152-3p F ACACTCCAGCTGGGTCAGTGCATGACAG

miR-152-3p R CTCAACTGGTGTCGTGGA

Accenssion ([MI0000253](http://www.mirbase.org/cgi-bin/mirna_entry.pl?acc=MI0000253))

miR-148a-3p F TCAGTGCACTACAGAACTTTGT

miR-148a-3p R TCTGTCAACGATACGCTACGT

Accenssion ([MI0000617](http://www.mirbase.org/cgi-bin/mirna_entry.pl?acc=MI0000617))

miR-148b-3p F   GCGTCAGTGCATCACAGAACTTTGT miR-148b-3p R CGAATTCTAGAGCTCGAGGCAGGCGACA

Accenssion (NR_138085)

U6 F GAGGGCCTATTTCCCATGATT

U6 R TAATTAGAATTAAT TTGACT

**Table S2**

shNC 5′-UUCUCCGAACGUGUCACGUAA3′;

shDNMT1 5-′GCGGCATGAACCGCTTCAATT3′;

siSOS1 5′-UUCUCCGAACGUUCACGUTT-3′.

SOS1 cDNA 5′-GCGGTAGGCGTGTACGGT-3′.

miR-152-3p inhibitor 5ʹ-CCAAGUUCUG UCAUGCACUGA-3ʹ;

miR-NC 5ʹ-GGAACUUAG CCACUGUGAAUU-3ʹ;

miR-152-3p

mimic 5ʹ-UR-CGTTCTCCTTGTCTTCTCTGCAUGACAGAACUUGG-3ʹ;

**Table S3**

# Antibody Catalog number Manufacturer Dilution

# α-Tubulin Mouse mAb ab7291 abcam 1:2000

# CD133 Rabbit mAb 51917 Cell Signaling 1:1000

# CD44 Mouse mAb 3570 Cell Signaling 1:1000

# DNMT1 Rabbit mAb 5032 Cell Signaling 1:1000

# SOS1 Rabbit mAb 12409 Cell Signaling 1:1000

# Oct-4 Antibody 2750 Cell Signaling 1:1000

Sox2 Rabbit mAb 3579 Cell Signaling 1:1000

**Table S4**

# Antibody Catalog number Manufacturer Dilution

# DNMT1 Rabbit mAb 5032 Cell Signaling 1:200

# CD44 Mouse mAb 3570 Cell Signaling 1:100

# SOS1 Rabbit mAb 55041-1-AP proteintech 1:200
